# Emergence of beta/gamma oscillations: ING, PING, and what about RING?

**DOI:** 10.1186/1471-2202-12-S1-P230

**Published:** 2011-07-18

**Authors:** Vasile V Moca, Raul C Mureşan

**Affiliations:** 1 Experimental and Theoretical Neuroscience, Center for Cognitive and Neural Studies, Romanian Institute of Science and Technology, Cluj-Napoca, 400487, Romania; 2 Department of Neurophysiology, Max-Planck Institute for Brain Research, Frankfurt am Main, 60528 Germany

## Background

Oscillatory activity in high-beta and gamma bands (20-80Hz) is known to play an important role in cortical processing being linked to cognitive processes and behavior. Beta/gamma oscillations are thought to emerge in local cortical circuits via two mechanisms: the interaction between excitatory principal cells and inhibitory interneurons – the pyramidal-interneuron gamma (PING) [1], and in networks of coupled inhibitory interneurons under tonic excitation – the interneuronal gamma (ING) [2]. Experimental evidence underlines the important role of inhibitory interneurons and especially of the fast spiking (FS) interneurons [3,4]. We show in simulation that an important property of FS neurons, namely the membrane resonance (frequency preference), represents an additional mechanism – the resonance induced gamma (RING), i.e. modulation of oscillatory discharge by resonance. RING promotes frequency stability and enables oscillations in purely excitatory networks.

## Methods

Local circuits were modeled with small world networks of 80% excitatory and 20% inhibitory neuron populations interconnected in small-world topology by realistic conductance-based synapses. Neuron populations were leaky integrate and fire (LIF) or Izhikevich resonator (RES) neurons. We also tested networks of purely inhibitory and purely excitatory RES neurons. Networks were stimulated with miniature postsynaptic potentials (MINIs) [5] and with low frequency sinusoidal (0.5 Hz) input that mimics the effect of gratings passing trough the visual field. The activity was calibrated to match recordings from cat visual cortex (firing rate, oscillatory activity).

## Results

Sinusoidal input modulates network oscillation frequency. This effect is most prominent in IF excitatory and IF inhibitory (IF-IF) networks and less prominent (about 4 times) in IF-RES or RES-IF networks where frequency remains relatively stable. The most stable frequency was observed in networks of pure resonators (RES-RES, None-RES, RES-None). Interestingly, purely excitatory RES networks (RES-None) were also able to exhibit oscillations through RING. By contrast purely excitatory or inhibitory IF networks (IF-None, None-IF) were not able to express oscillations under these conditions, matching experimental parameters.

## Conclusions

In both PING and ING, adding membrane resonance to principal cells or inhibitory interneurons stabilizes network oscillation frequency via the RING mechanism. Notably, in networks of purely excitatory networks, where ING and PING are not defined, oscillations can emerge via the RING mechanism if membrane resonance is expressed. Thus, RING appears as a potentially important mechanism for promoting stable network oscillations.
